# Prediction formulae of sagittal alignment in thoracolumbar kyphosis secondary to ankylosing spondylitis after osteotomy

**DOI:** 10.1038/s41598-023-34475-3

**Published:** 2023-05-12

**Authors:** Jianzhou Luo, Zili Yang, Chunguang Duan, Xujiao Feng, Lei Tan, Yanzhe Wei, Li Jiang, Tailin Wu

**Affiliations:** 1 Health Science Center, Shenzhen University, Shenzhen, 518000 Guangdong People’s Republic of China; 2 Department of Orthopedics, Shenzhen University General Hospital, Shenzhen, 518000 Guangdong People’s Republic of China; 3 Shenzhen Institutes of Advanced Technology, Chinese Academy of Sciences, Shenzhen, 518000 Guangdong People’s Republic of China; 4 School of Life Science and Technology, Xi’an Jiaotong University, Xi’an, 710049 Shaanxi People’s Republic of China; 5 Shenzhen Key Laboratory of Precision Medicine for Hematological Malignancies, Shenzhen University General Hospital, Shenzhen, 518000 Guangdong People’s Republic of China

**Keywords:** Skeleton, Rheumatoid arthritis, Orthopaedics, Whole body imaging

## Abstract

To construct and validate prediction formulae of sagittal alignment in thoracolumbar kyphosis secondary to ankylosing spondylitis (AS) after osteotomy. A total of 115 AS patients who suffered from thoracolumbar kyphosis and underwent osteotomy were enrolled, with 85 patients in derivation group and 30 patients in validation group. Radiographic parameters were measured on lateral radiographs, including thoracic kyphosis, lumbar lordosis (LL), T1 pelvic angel (TPA), sagittal vertical axis (SVA), osteotomized vertebral angle, pelvic incidence (PI), pelvic tilt (PT), sacral slope (SS), and PI and LL mismatch (PI-LL). Prediction formulae of SS, PT, TPA and SVA were established; and their effectiveness was evaluated. There was no significant difference in baseline characteristics between the two groups (*p* > 0.05). In derivation group, LL and PI-LL were correlated with SS, and were then used to establish the prediction formula of SS[SS = − 12.791–0.765 × (LL) + 0.357 × (PI-LL), R^2^ = 68.3%]; PI and PI-LL were correlated with PT, and the prediction formula of PT were thus established[PT = 12.108 + 0.402 × (PI-LL) + 0.252 × (PI), R^2^ = 56.8%]; PT, PI-LL and LL were correlated with TPA, and were used to establish the prediction formula of TPA[TPA = 0.225 + 0.597 × (PT) + 0.464 × (PI-LL)-0.161 × (LL), R^2^ = 87.4%]; PT, PI-LL and age were correlated with SVA, and were used to establish the prediction formula of SVA[SVA = 36.157 + 2.790 × (PI-LL) + 1.657 × (Age)-1.813 × (PT), R^2^ = 41.5%]. In validation group, the predictive SS, PT, TPA and SVA were basically consistent with corresponding real values; and the mean error between predictive values and real values was of 1.3° in SS, 1.2° in PT, 1.1° in TPA and 8.6 mm in SVA. Postoperative SS, PT, TPA and SVA could be predicted with PI and the planned LL and PI-LL using prediction formulae, providing a method for AS kyphosis to plan postoperative sagittal alignment. Change of pelvic posture after osteotomy was quantitatively evaluated using the formulae.

## Introduction

Sagittal malalignment causes internal fixation failure and low health-related quality of life in ankylosing spondylitis (AS) after osteotomy^1–4^. The causes of sagittal malalignment are mainly linked to inadequate kyphosis correction and mismatched spinopelvic harmony^5–7^. Reconstructing balanced sagittal alignment and harmonious spinopelvic relationship are necessary to maintain kyphosis correction and achieve satisfactory clinical outcome in AS patients^8,9^. In our previous study^10^, the risk factors of sagittal malalignment after osteotomy were evaluated, and the postoperative immediate sagittal vertical axis (SVA) of ≤ 74 mm was recommended for preventing sagittal imbalance. However, given the complex interaction compensatory mechanism between spine and pelvis, how to achieve SVA of ≤ 74 mm has not been established. Generally, postoperative sagittal alignment is constructed by surgeon according to the plan. And an appropriate surgical plan is the key step to construct a proper sagittal alignment. Predicting sagittal alignment and knowing the required degree of osteotomy can be of great help in designing a successful surgical plan. However, there is no clear method to predict the postoperative sagittal alignment for AS kyphosis, especially to evaluate the change of pelvic posture after osteotomy^11–13^, which might hamper the development of an appropriate surgical plan. In adult spinal deformity (ASD), Lafage et al.^14,15^ proposed two formulae to predict the postoperative pelvic tilt (PT) and SVA, and guide the formulation of surgical plans, which contributed to resolving the problem in ASD. However, whether the sagittal alignment of AS kyphosis after osteotomy could be predicted and used for devising a surgical plan remains unclear.

Therefore, in this study, we aimed to construct and validate prediction formulae for predicting pelvic posture [PT, sacral slope (SS)] and global alignment [T1 pelvic angel (TPA), SVA] in thoracolumbar kyphosis secondary to AS after osteotomy; and proposed a novel method to devise surgical plans for these patients.

## Materials and methods

### Subjects and study design

Consecutive AS thoracolumbar kyphosis cases who underwent osteotomy between January 2010 and January 2020, were retrospectively reviewed. Anteroposterior and lateral radiographs of whole spine were obtained in all patients, who were instructed to stand in a freestanding position with elbows flexed at approximately 45° and fingertips under the chin. This position allowed for consistent technique and comparative analysis^11,16,17^. Indications for surgery were that: (1) thoracolumbar kyphosis with global kyphosis > 50°, SVA > 50 mm or PT > 25°, (2) impaired quality of life, including having trouble in walking upright, lying flat and seeing horizontally, and (3) strong desire for surgical treatment. Patients aged ≥ 18 years and with osteotomy on thoracolumbar/lumbar vertebrae were enrolled in this study. Those with hip or knee contracture or ankylosis, postoperative pseudarthrosis or instrumentation failure during the follow-up were excluded. Finally, a total of 115 AS patients met the criteria and were randomly divided into two groups, with 85 patients in derivation group and 30 patients in validation group. The data from derivation group was used for exploring and developing prediction formulae, including analyzing correlation between age, spinal and pelvic parameters, and establishing prediction formulae of postoperative SS, PT, TPA and SVA. The data from validation group was used for assessing and validating the formulae, including evaluating reliability of prediction formulae, and comparing difference between predictive value and real value.

### Data collection

**Figure 1:**
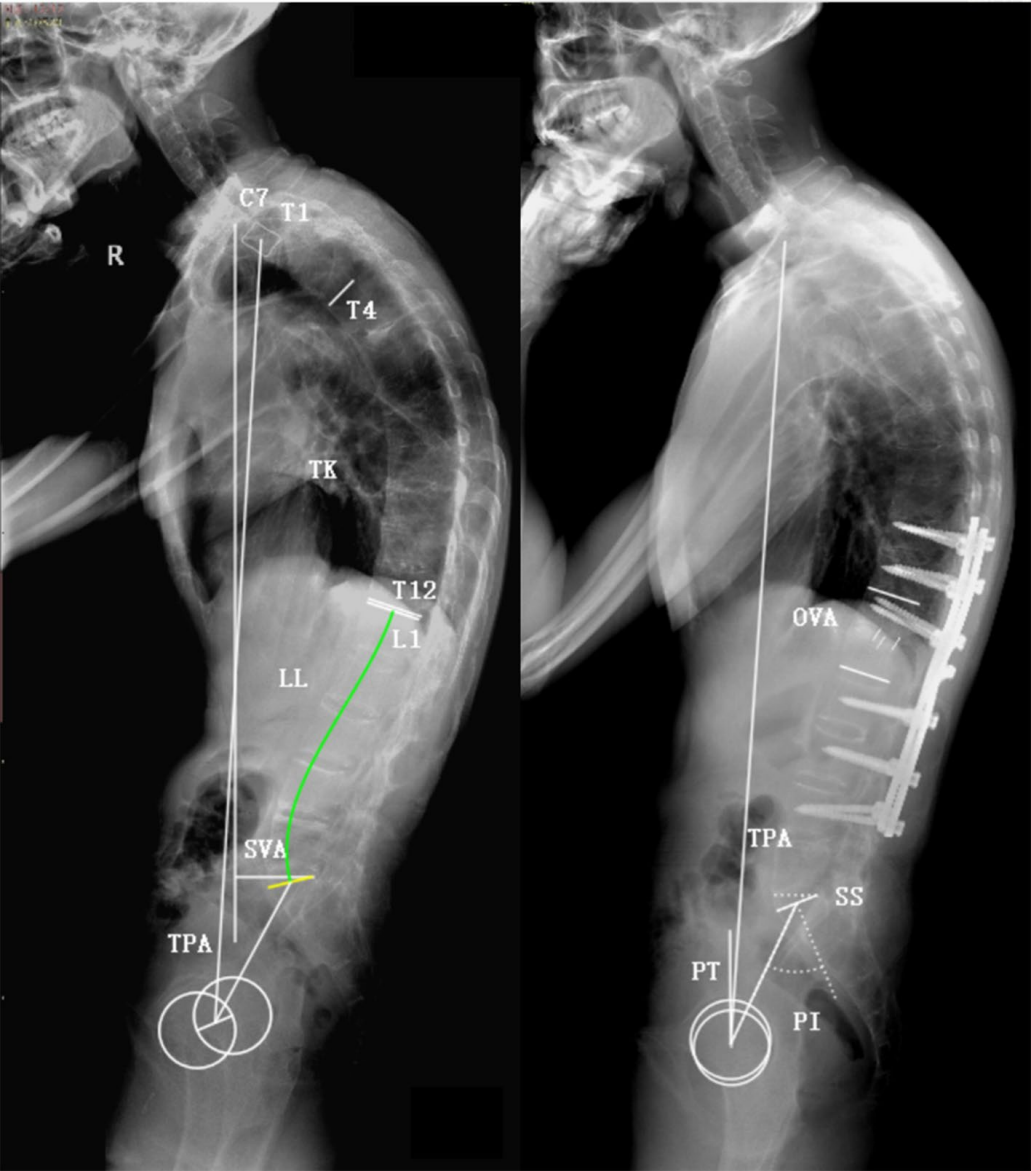
Illustration of radiographic parameters measurement. Thoracic kyphosis (TK): the Cobb angle from the T4 upper endplate to the T12 lower endplate; lumbar lordosis (LL): the Cobb angle from the L1 upper endplate to the S1 upper endplate; T1 pelvic angle (TPA): the angle between a line joining the center of T1 and the femoral head axis and a line from the center of the femoral head axis to the midpoint of the S1 upper endplate; sagittal vertical axis (SVA): the distance between the C7 plumb line and the posterosuperior corner of S1; osteotomized vertical angle (OVA): the angle between the lower endplate of the osteotomized vertebra and the upper endplate of the cranial adjacent vertebra; pelvic incidence (PI) : the angle between the line perpendicular to the S1 upper endplate and the line from the center of the S1 upper endplate to the center of the femoral head axis; pelvic tilt (PT): the angle between the vertical line and the line from the center of the S1 upper endplate to the center of the femoral head axis; sacral slope (SS): the angle between the S1 upper endplate and the horizontal line; PI and LL mismatch (PI-LL): the pelvic incidence value minus the lumbar lordosis value.

Standing anteroposterior and lateral radiographs of the whole spine were obtained preoperatively and postoperatively (6 months after surgery). The radiographic parameters were measured on lateral radiographs using Surgimap Spine Software (version 2.3.1.3, Nemaris Inc., New York, USA), including thoracic kyphosis (TK), lumbar lordosis (LL), TPA, SVA, osteotomized vertebral angle (OVA) (for two-level osteotomy, OVA was defined as the sum of two Cobb angles measured in two osteotomized vertebrae), pelvic incidence (PI), PT, SS, and PI and LL mismatch (PI-LL) (Fig. 1). All parameters were measured independently by two experienced professionals. PI was considered as a fixed parameter before and after surgery in this study^18,19^. The demographic and surgical data was collected and recorded for all patients.

### Statistical analysis

Measurement data were expressed as the mean ± standard deviation. Statistical analysis was performed using SPSS software (version 22.0, SPSS, Inc., Chicago, IL). Baseline characteristics of demographic and radiographic data in two groups were compared using two independent samples t-tests and chi-square tests. Correlation between age, spinal and pelvic parameters were determined using Pearson’s coefficient analysis. Based on correlation between target parameters (SS, PT, TPA and SVA) and the surgically controllable parameters (LL and PI-LL), prediction formulae were then established using multiple stepwise linear regression analysis. Differences between predictive values and real values were evaluated using paired t-tests in validation group. Correlation between osteotomy parameters (sites of osteotomy and OVA correction) and the correction of target parameters were analyzed using Spearman (for sites of osteotomy) and Pearson (for OVA corrections) coefficients analysis. Effect of different sites of osteotomy on the correction of target parameters was determined using analysis of variance. A difference with a *p*-value < 0.05 was considered statistically significant.

### Ethical approval

The study was performed in accordance with the Declaration of Helsinki, and approved by the institutional review board of Shenzhen University General Hospital. The informed consent was obtained from all participants for both study participation and publication of identifying information/images in an online open-access publication.

## Results

### Comparison of baseline characteristics between two groups

A total of 115 AS patients (102 men and 13 women) with an average age of 37.3 ± 8.1 years (range, 20–64 years) were enrolled, including 85 in derivation group and 30 in validation group. Eighty-nine patients underwent one-level osteotomy and 26 patients underwent two-level osteotomy. There was no significant difference in demographic and radiographic data between the derivation group and the validation group (all *p* > 0.05) (Table 1).

**Table 1:** Comparison of baseline characteristics between the derivation and validation groups.

Variables	Total	Derivation group	Validation group	*P* value
Age (years)	37.3 ± 8.1	37.1 ± 8.8	37.9 ± 6.3	0.605
Sex (men/women)	102/13	75/10	27/3	0.701
No. of one/two-level osteotomy	89/26	63/22	26/4	1.000
Sites of one-level osteotomy: T12/L1/L2/L3/L5	1/19/54/14/1	1/16/34/11/1	0/3/20/3/0	
Sites of two-level osteotomy:	1/1/2/11/3/4/1/3	1/1/1/8/3/4/1/3	0/0/1/3/0/0/0/0	
T10 and L1/ T11 and L3/				
T12 and L2/ T12 and L3/				
L1 and L3/ L1 and L4/				
L1 and L5/ L2 and L5				
Preoperative TK (°)	53.7 ± 15.8	54.7 ± 16.5	51.2 ± 13.6	0.298
Preoperative LL (°)	5.6 ± 21.0	5.7 ± 21.7	4.7 ± 19.4	0.817
Preoperative PT (°)	36.9 ± 12.1	38.3 ± 11.2	34.3 ± 12.2	0.101
Preoperative PI (°)	47.0 ± 12.0	48.2 ± 12.7	44.4 ± 9.1	0.136
Preoperative SS (°)	10.3 ± 11.8	10.1 ± 12.2	10.2 ± 10.2	0.982
Preoperative PI-LL (°)	52.3 ± 21.2	53.3 ± 21.9	49.4 ± 19.8	0.396
Preoperative TPA (°)	56.0 ± 18.2	57.1 ± 19.3	53.1 ± 15.1	0.322
Preoperative SVA (mm)	234.3 ± 93.5	227.1 ± 90.4	247.1 ± 96.2	0.309
Postoperative TK (°)	48.5 ± 14.0	49.3 ± 14.4	46.9 ± 12.3	0.410
Postoperative LL (°)	− 32.3 ± 17.6	− 32.0 ± 18.2	− 34.4 ± 14.8	0.520
Postoperative PT (°)	29.3 ± 10.4	30.6 ± 10.4	25.8 ± 9.9	0.051
Postoperative PI (°)	46.7 ± 11.1	47.8 ± 11.4	43.8 ± 9.9	0.088
Postoperative SS (°)	17.4 ± 11.9	16.9 ± 12.8	18.3 ± 8.9	0.600
Postoperative PI-LL (°)	14.3 ± 16.6	16.0 ± 16.6	9.7 ± 16.0	0.076
Postoperative TPA (°)	29.9 ± 11.5	31.0 ± 11.9	26.8 ± 9.6	0.081
Postoperative SVA (mm)	87.3 ± 58.9	87.1 ± 58.4	87.9 ± 61.5	0.950
OVA correction (°)	45.8 ± 18.4	45.8 ± 19.9	46.0 ± 14.0	0.950

Negative number represents lordosis, positive number represents kyphosis.

*TK* thoracic kyphosis, *LL* lumbar lordosis, *PT* pelvic tilt, *PI* pelvic incidence, *SS* sacral slope, *PI-LL* PI and LL mismatch, *TPA* T1 pelvic angel, *SVA* sagittal vertical axis, *OVA* osteotomized vertical angle.

### Correlation between age, spinal and pelvic parameters

The postoperative SS was strongly correlated with LL, PI and PI-LL (r = − 0.732, 0.595 and − 0.397, respectively, *p* < 0.05); the postoperative PT was significantly correlated with LL, PI and PI-LL (r = 0.380, 0.433 and 0.711, respectively, *p* < 0.05); the postoperative TPA was significantly correlated with LL, PT, PI, SS, PI-LL and age (r = 0.450, 0.867, 0.467, − 0.290, 0.826 and 0.223, respectively, *p* < 0.05); and the postoperative SVA was significantly correlated with LL, PT, PI, PI-LL and age (r = 0.357, 0.281, 0.265, 0.588 and 0.261, respectively, *p* < 0.05) (Table 2).

**Table 2:** Correlation between age, spinal and pelvic parameters.

Variables	TK	LL	PT	PI	SS	PI-LL	TPA	SVA	Age
TK	1								
LL	− 0.434^†^	1							
PT	0.076	0.380^†^	1						
PI	0.126	− 0.423^†^	0.433^†^	1					
SS	0.047	− 0.732^†^	− 0.452^†^	0.595^†^	1				
PI-LL	− 0.351^†^	0.771^†^	0.711^†^	0.242*	− 0.397^†^	1			
TPA	0.116	0.450^†^	0.867^†^	0.476^†^	− 0.290^†^	0.826^†^	1		
SVA	0.140	0.357^†^	0.281*	0.265*	0.021	0.588^†^	0.673^†^	1	
Age	0.281^†^	− 0.164	0.195	0.370^†^	− 0.181	0.083	0.223*	0.261*	1

*TK* thoracic kyphosis, *LL* lumbar lordosis, *PT* pelvic tilt, *PI* pelvic incidence, *SS* sacral slope, *PI-LL* PI and LL mismatch, *TPA* T1 pelvic angel, *SVA* sagittal vertical axis.

*Indicates a statistically significant correlation (*p* < 0.05).

^†^Indicates a statistically significant correlation (*p* < 0.01).

### Prediction formulae of postoperative SS, PT, TPA and SVA

Based on the correlation between SS, PT, TPA and SVA, and other postoperative parameters, the prediction formulae of SS, PT, TPA and SVA were then established using multiple stepwise linear regression analysis.

The LL, PI and PI-LL were entered into analysis, after which only the LL and PI-LL were included in the model. The prediction formula of SS was thus established: SS = − 12.791–0.765 × (LL) + 0.357 × (PI-LL), *p* < 0.001, with adjusted R^2^ = 68.3% (Table 3).

**Table 3:** Multiple stepwise regression analysis for prediction formulas of SS, PT, TPA and SVA.

Model	Variables	Unstandardized coefficients		Standardized coefficients	T	*P* value	VIF	Adjusted R^2^
		B	Standard error	Beta				
SS	(Constant)	− 12.791	3.102	−	− 4.123	< 0.001	–	68.3%
	LL	− 0.765	0.064	− 1.146	− 11.899	< 0.001	2.463	
	PI-LL	0.357	0.071	0.487	5.054	< 0.001	2.463	
PT	(Constant)	12.108	3.206	–	3.777	< 0.001	–	56.8%
	PI-LL	0.402	0.046	0.644	8.717	< 0.001	1.062	
	PI	0.252	0.067	0.277	3.751	< 0.001	1.062	
TPA	(Constant)	0.225	2.133	–	0.105	0.916	–	87.4%
	PT	0.597	0.071	0.519	8.452	< 0.001	2.431	
	PI-LL	0.464	0.065	0.642	7.147	< 0.001	5.207	
	LL	− 0.161	0.044	− 0.245	− 3.616	0.001	2.969	
SVA	(Constant)	36.157	24.917	–	1.451	0.151	–	41.5%
	PI-LL	2.790	0.427	0.790	6.533	< 0.001	2.001	
	Age	1.657	0.580	0.250	2.857	0.005	1.051	
	PT	− 1.813	0.693	− 0.322	− 2.617	0.011	2.072	

*SS* sacral slope, *LL* lumbar lordosis, *PI* pelvic incidence, *PI-LL* PI and LL mismatch, *PT* pelvic tilt, *TPA* T1 pelvic angel, *SVA* sagittal vertical axis.

Model SS: Durbin-Watson = 1.939; Model PT: Durbin-Watson = 1.921; Model TPA: Durbin-Watson = 2.019; Model SVA: Durbin-Watson = 1.970.

The LL, PI and PI-LL were entered into analysis, and only the PI and PI-LL were included in the model. The prediction formula of PT was thus established: PT = 12.108 + 0.402 × (PI-LL) + 0.252 × (PI), *p* < 0.001, with adjusted R^2^ = 56.8% (Table 3).

The LL, PT, PI, SS, PI-LL and age were entered into analysis, after which the PT, PI-LL and LL were included in the model. The prediction formula of TPA was thus established: TPA = 0.225 + 0.597 × (PT) + 0.464 × (PI-LL)-0.161 × (LL), *p* < 0.001, with adjusted R^2^ = 87.4% (Table 3).

The LL, PT, PI, PI-LL and age were entered into analysis, after which the PI-LL, PT and age were included in the model. The prediction formula of SVA was thus established: SVA = 36.157 + 2.790 × (PI-LL) + 1.657 × (Age)-1.813 × (PT), *p* < 0.001, with adjusted R^2^ = 41.5% (Table 3).

### Validation of the prediction formulae

**Figure 2:**
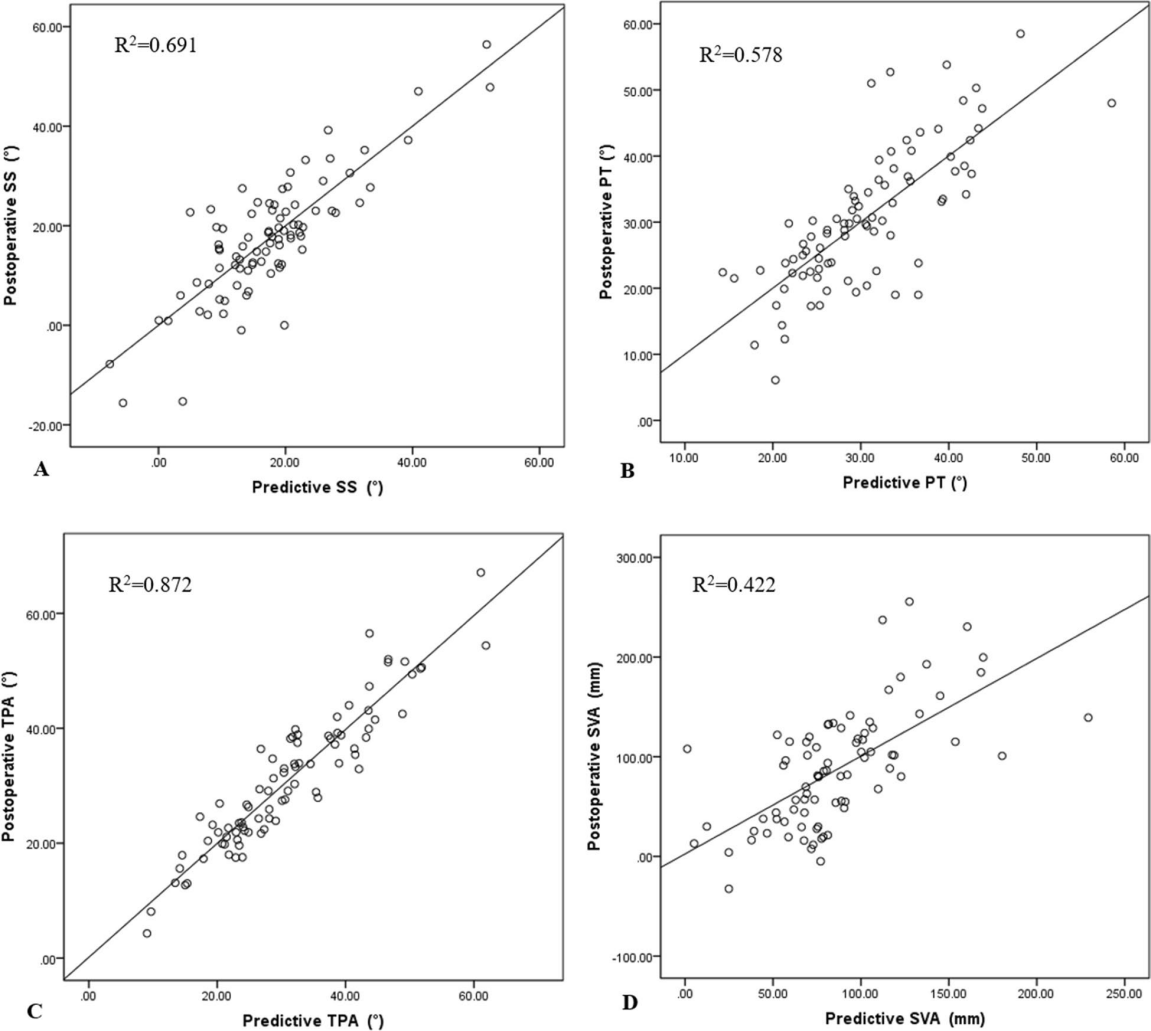
Scatter diagram demonstrated the relationship between predictive values and real values in SS (**A**), PT (**B**), TPA(**C**) and SVA (**D**) in derivation group.

**Figure 3:**
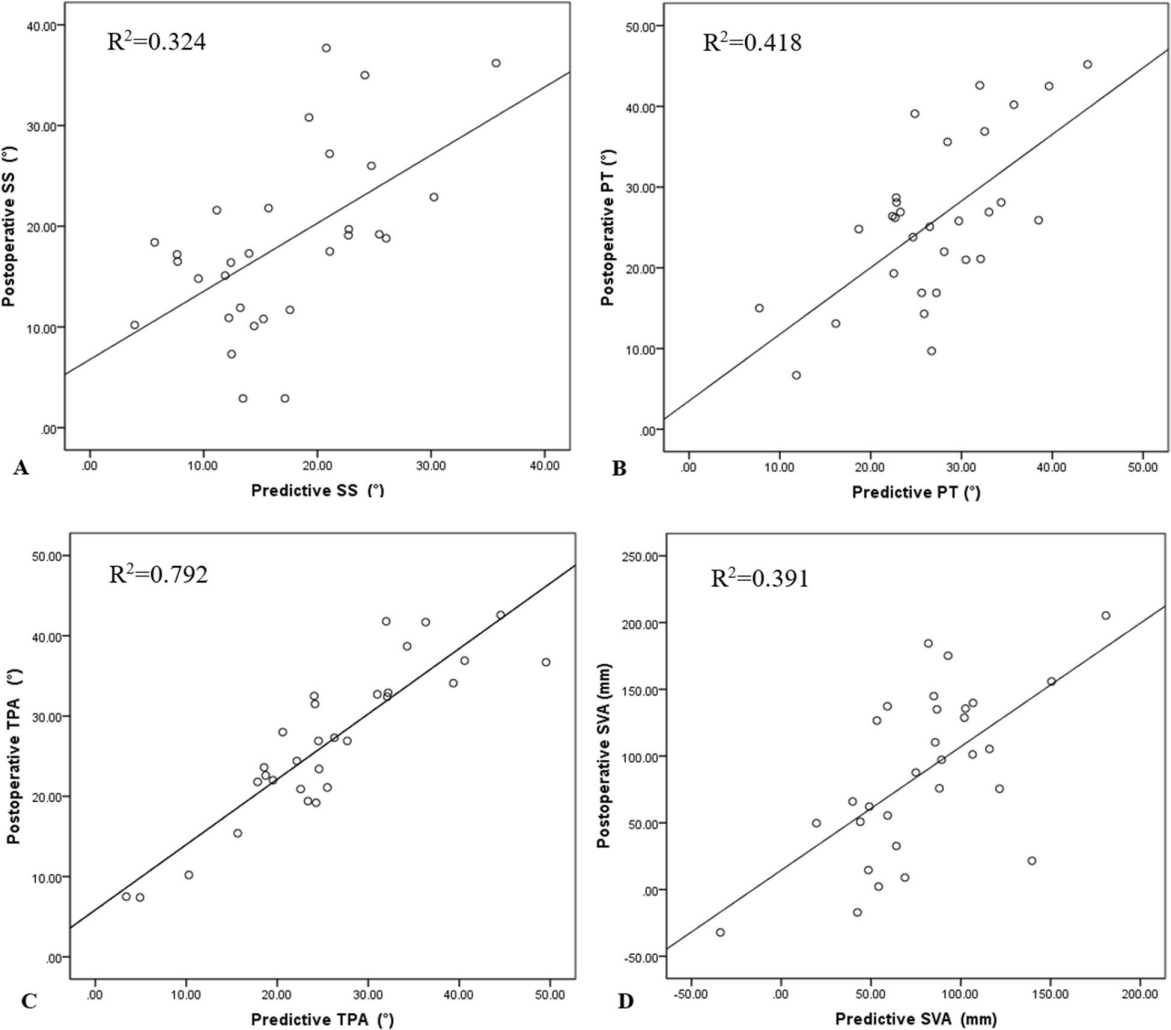
Scatter diagram demonstrated the relationship between predictive values and real values in SS (**A**), PT (**B**), TPA(**C**) and SVA (**D**) in validation group.

In derivation group, the predictive SS, PT, TPA and SVA which were calculated using the prediction formulae with postoperative PI, PI-LL and LL (surgically controllable parameters), were basically in agreement with the postoperative real values (R^2^ = 69.1%, 57.8%, 87.2% and 42.2%, respectively, Fig. 2A–D). The data from validation group confirmed that, the postoperative SS, PT, TPA and SVA could be predicted with postoperative PI, PI-LL and LL through the prediction formulae (R^2^ = 32.4%, 41.8%, 79.2% and 39.1%, respectively, Fig. 3A–D), and the mean error between the predictive values and the real values was of 1.3 ± 7.7° in SS, 1.2 ± 7.7° in PT, 1.1 ± 4.8° in TPA and 8.6 ± 48.6 mm in SVA (Table 4).

**Table 4:** Comparison of predictive value and real value in validation group (n = 30).

Parameters	Real value	Predictive value	Difference	*P* value	95%CI	
					lower	upper
SS (°)	18.3 ± 8.9	17.0 ± 7.5	1.3 ± 7. 7	0.367	− 1.58	4.15
PT (°)	25.8 ± 9.9	27.0 ± 7.8	1.2 ± 7.7	0.397	− 4.08	1.67
TPA (°)	26.8 ± 9.6	25.7 ± 10.5	1.1 ± 4.8	0.233	− 0.72	2.86
SVA (mm)	87.9 ± 61.5	79.3 ± 41.6	8.6 ± 48.6	0.337	− 9.37	26.52

Real value was measured on the radiographs, predictive value was calculated using the prediction formulae.

*SS* sacral slope, *PT* pelvic tilt, *TPA* T1 pelvic angel, *SVA* sagittal vertical axis, *CI* confidence interval.

### Effects of different osteotomy sites on SS, PT, TPA and SVA correction

Eighty-six cases of AS kyphosis who underwent one-level osteotomy at L1, L2 and L3 were included for analyzing the effect of osteotomized sites on pelvic posture and global alignment. Sites of osteotomy were correlated with SVA correction (r = 0.307, *p* = 0.004); and the OVA correction was correlated with TPA and SVA correction (r = 0.697 and 0.365, respectively, *p* < 0.05). But both the osteotomy sites and OVA correction were not statistically correlated with the correction of SS and PT (*p* > 0.05) (Table 5). There was no significant difference in the corrections of OVA, SS, PT and TPA at different sites of osteotomy (*p* > 0.05, Table 6). The SVA correction achieved at L2 (*p* = 0.029) and L3 (*p* = 0.068) osteotomy was larger than that achieved at L1 osteotomy (Table 6).

**Table 5:** Correlation between osteotomy parameters and the correction of SS, PT, TPA and SVA.

Measurement	Sites of osteotomy	OVA correction (°)
SS correction (°)	− 0.047	− 0.014
PT correction (°)	− 0.002	0.075
TPA correction (°)	0.206	0.697*
SVA correction (mm)	0.307*	0.365*

*SS* sacral slope, *PT* pelvic tilt, *TPA* T1 pelvic angel, *SVA* sagittal vertical axis.

*Indicates a statistically significant correlation (*p* < 0.05).

**Table 6:** Difference of different osteotomy sites on SS, PT, TPA and SVA correction.

Level of osteotomy	No. of patients	OVA correction (°)	SS correction (°)	PT correction (°)	TPA correction (°)	SVA correction (mm)
L1	18	36.6 ± 16.0	8.1 ± 6.2	8.2 ± 7.4	20.3 ± 9.0	88.0 ± 56.0*
L2	54	37.6 ± 11.0	8.0 ± 9.0	8.1 ± 8.5	23.5 ± 10.7	130.6 ± 58.9*
L3	14	43.0 ± 13.5	5.6 ± 6.8	6.4 ± 10.2	28.7 ± 13.2	165.8 ± 106.9

*OVA* osteotomized vertebral angle, *SS* sacral slope, *PT* pelvic tilt, *TPA* T1 pelvic angel, *SVA* sagittal vertical axis.

*Indicates a statistically significant difference between L1 and L2 (*p* < 0.05).

### Case illustration for surgical plan

**Figure 4:**
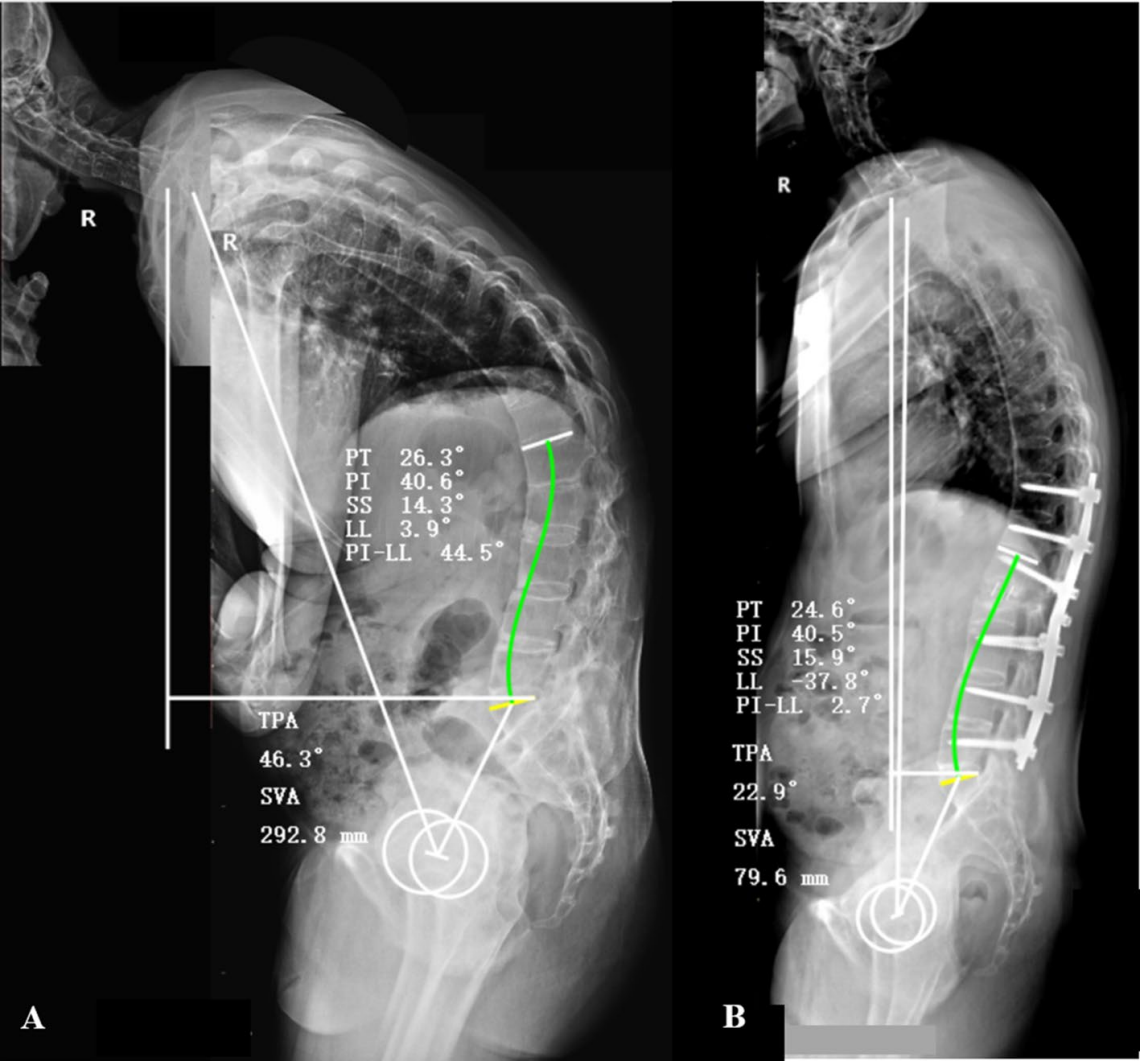
A 42-year-old man suffered from thoracolumbar kyphosis secondary to ankylosing spondylitis. (**A**) Preoperative radiographs showed severe global kyphosis and sagittal imbalance. (**B**) The patient underwent one-level three-column osteotomy on L2, following which the kyphosis was corrected and the sagittal alignment was restored.

Predictive ability of the formulae could be applied in surgical plan. The planned PI-LL in regional curvatures achieved by osteotomy, along with the fixed PI, were used to predict the postoperative SS, PT, TPA and SVA. The PI (40.5°) was the only parameter that required to be measured in preoperative radiograph (Fig. 4). With the planned PI-LL (3.0°), the LL was calculated using equation of LL = PI-3.0 = 37.5°. The planned PI-LL and LL were then used to predict SS and PT using the formulae. Subsequently, the TPA and SVA were then predicted with the predictive PT and age (42). The detail of calculation was as follows:

$${\text{SS}} = - {12}.{791} - 0.{765} \times \left( { - {37}.{5}} \right) + 0.{357} \times \left( {{3}.0} \right) = {17}.0^\circ ,$$

$${\text{PT}} = {12}.{1}0{8} + 0.{4}0{2} \times \left( {{3}.0} \right) + 0.{252} \times \left( {{4}0.{5}} \right) = {23}.{5}^\circ ,$$

$${\text{TPA}} = 0.{225} + 0.{597} \times \left( {{23}.{5}} \right) + 0.{464} \times \left( {{3}.0} \right) - 0.{161} \times ( - {37}.{5}) = {21}.{7}^\circ ,$$

and

$${\text{SVA}} = {36}.{157} + {2}.{79}0 \times \left( {{3}.0} \right) + {1}.{657} \times \left( {{42}} \right) - {1}.{813} \times \left( {{23}.{5}} \right) = {71}.{\text{5 mm}}.$$

Analysis of the results indicated that the predictive SS, PT and TPA were similar to the postoperative real values (Table 7, Fig. 4). The postoperative real SVA was 8.1 mm larger than the predictive SVA, and it would be closer to the predictive value with lower site of osteotomy.

**Table 7:** Case illustration of prediction formulae for application.

Variables	Preoperative	Planned	Postoperative
PI (°)	40.5	40.5	40.5
LL (°)	3.3	− 37.5	− 37.8
PI-LL (°)	43.8	3.0	2.7
SS (°)	14.8	17.0	15.9
PT (°)	25.7	23.5	24.6
TPA (°)	46.3	21.7	22.9
SVA (mm)	289.2	71.5	79.6
Age (year)	42	42	42

*PI* pelvic incidence, *LL* lumbar lordosis, *PI-LL* PI and LL mismatch, *SS* sacral slope, *PT* pelvic tilt, *TPA* T1 pelvic angel. *SVA* sagittal vertical axis.

For LL, negative number represents lordosis, positive number represents kyphosis.

## Discussion

Sagittal malalignment following osteotomy is a major determinant of clinical outcome in AS patients^2,6^. Given the complex interactional compensatory mechanisms between spine and pelvis, it is difficult for the surgeon to control and correct sagittal malalignment, especially to restore the neutral pelvic position^11,13^. Moreover, the retroversion of pelvis cannot be corrected directly, and is restored mainly by reconstructing lumbar lordosis and spinopelvic harmony^12,20^. However, the correlation between postoperative sagittal alignment and compensatory pelvic rotation is unclear, and the extent to which sagittal alignment should be reconstructed after osteotomy to compensate for pelvic rotation remains unknown.

In this study, the pelvic parameters (SS and PT) were significantly correlated with LL, PI and PI-LL; and the predictive formulae of SS and PT were then established based on the correlation. The formulae make it possible to evaluate the extent of sagittal alignment reconstruction to compensate for postoperative pelvic rotation. Using the formulae, the predictive SS and PT could be estimated with the fixed PI and the planned PI-LL and LL (surgically controllable parameters). Subsequently, the change of pelvic posture after osteotomy was predicted, which was reflected by the correction of SS and PT. Notably, the formulae revealed that the key to restoring pelvic position was to construct lumbar lordosis (LL) and correct spinopelvic harmony (PI-LL), which was consistent with the conclusions of Schwab^21^ and Liu^22^. The accurately predicted postoperative pelvic parameters (SS and PT) play an important role in devising proper and individualized plan. For example, the predictive SS could be used in the ASKyphoplan^23^ for devising individualized plan according to the individual pelvic pattern, instead of using the same angle of 40° (normal range) for every patient^24^. The PT prediction formula of AS kyphosis was particularly suitable for Song’s method^17^ to devise a personalized plan based on the specificity of disease, instead of using the prediction formula of PT = − 7 + 0.37 × PI in asymptomatic subjects^19^. Although PT could be calculated with PI and predictive SS using the formula of PT = PI-SS^25^, it might include some calculation error by using predictive SS for further predicting PT. Hence, it would be more reliable to use the original PI and planned PI-LL to predict postoperative PT through the prediction formula instead of PI-predictive SS, which was also proven by our data (difference between the predictive PT calculated by our prediction formula and the one calculated by PI-predictive SS was 0.4°, *p* < 0.001).

The global alignment parameters (TPA and SVA) were significantly correlated with LL, PT, PI, PI-LL and age. The prediction formulae of TPA and SVA were then established. With the predictive PT and the planned PI-LL and LL, the postoperative TPA and SVA were calculated using the formulae. Subsequently, the aforementioned postoperative immediate SVA of ≤ 74 mm was thus predicted and applied for preventing sagittal imbalance^10^. The capability of the prediction formulae to predict TPA and SVA was different. The prediction formulae of TPA to predict postoperative TPA was more precise and reliable than the prediction formulae of SVA to predict postoperative SVA (adjusted R^2^: 87.4% vs. 41.5%). It was possibly because TPA was less affected by patient’s standing posture, and was more accurate than SVA in reflecting global alignment. Therefore, the TPA could be a better choice for the reference of assessing postoperative global alignment in surgical planning.

In this study, there was no significant correlation between the sites of osteotomy and the correction of SS and PT, which indicated that the sites of osteotomy might not affect the pelvis rotation postoperatively, but were positively correlated with SVA correction. The results were in contrast to Lafage^26^, who reported that the sites of osteotomy were correlated with changes of SS and PT, but not with the change of SVA in ASD. The contrasting results might be due to the different pathogenic processes between AS and ASD. In AS kyphosis, the sagittal alignment were stiff and fused, without pelvic version accommodation; the change of pelvic posture (SS and PT) was mainly achieved by using femoral head as fulcrum and rotating anteriorly. With a similar OVA correction, the lower site of osteotomy was selected, and the larger correction of global alignment (TPA and SVA) was achieved in AS kyphosis^24,27,28^. However, in ASD, the sagittal alignment was flexible and showed dynamic change, leaving variable modifications to SS, PT, TPA and SVA after osteotomy.

In the past, it was common to plan a surgery by calculating required osteotomized vertebral angle^24,28,29^. Ondra et al.^28^ proposed a mathematical method to calculate the osteotomized vertebral angle for correcting the kyphosis; but they failed to consider the pelvis compensation in their method. Van Royen et al.^24^ assumed an SS of 40° (normal range) as postoperative pelvis position, and then calculated the required osteotomized angle by trigonometric method. Obviously, the assumption of SS = 40° was not an appropriate angle for devising an individualized plan. Zheng^30^ and Song^17^ considered the pelvic compensation and used the theoretical PT as reference for plan; however, not all AS patients could totally compensate the PT to a theoretical value. The present study provided a novel method for devising individualized plan for AS kyphosis using the prediction formulae, which considered the correlation between pelvis compensation and sagittal alignment. In this method, planning proper target of PI-LL and performing it strictly were the key steps for successful radiographic outcome. PI-LL reflected spinopelvic harmony, and was linked to sagittal balance. The optimal PI-LL of ± 9 was recommended for the young, but some adjustments may be needed for older adults^21,31^. Lafage and colleagues^14,15^ used the prediction formulae of PT and SVA to devise a surgical plan for ASD, and obtained satisfactory radiographic outcome. Similarly, the prediction formulae of SS, PT, TPA and SVA in this study were a potentially useful method to devise individualized plan for AS kyphosis.

### Limitations

First, the planning method was performed using planned LL and PI-LL to predict postoperative sagittal alignment, which was only available to those with thoracolumbar/lumbar vertebrae osteotomy, but might be unsuitable for those with thoracic vertebrae osteotomy. For patients with ankylosed cervical vertebrae, PI-LL should be carefully and conservatively planned to ensure horizon vision and avoid over correction postoperatively. Second, the prediction formulae were applicable to patients with complete pelvis compensatory capacity; those with hip or knee soft-tissue contracture or ankylosis might lose pelvic compensatory ability and were unsuitable for the prediction formulae. Third, the effect of two-level osteotomy on sagittal alignment has not been analyzed for limited samples. In the future, a prospective and multi-center study with a larger sample size is required to confirm the conclusions.

## Conclusions

Postoperative SS, PT, TPA and SVA could be predicted with the fixed PI and the planned LL and PI-LL using prediction formulae, providing a method for AS kyphosis to plan postoperative sagittal alignment. Change of pelvic posture after osteotomy was quantitatively evaluated with the formulae. Selection of vertebral sites of osteotomy affected the global alignment correction but not the pelvis rotation.

## Data Availability

The datasets used and/or analyzed during the current study available from the corresponding author on reasonable request.
